# Upstaging of Tuberculosis in the Post-COVID-19 Era: A Case Series

**DOI:** 10.7759/cureus.54687

**Published:** 2024-02-22

**Authors:** Neeru Malik, Meenakshi Sidhar, Nidhi Prabha Sehgal, Anurag Gupta, Ishita Gupta

**Affiliations:** 1 Obstetrics and Gynaecology, Dr. Baba Saheb Ambedkar Medical College and Hospital, New Delhi, IND; 2 Pathology, Dr. Baba Saheb Ambedkar Medical College and Hospital, New Delhi, IND; 3 Anaesthesiology and Critical Care, Dr. Baba Saheb Ambedkar Medical College and Hospital, New Delhi, IND; 4 Pathology, University College of Medical Sciences and Guru Teg Bahadur (GTB) Hospital, New Delhi, IND

**Keywords:** female genital tuberculosis, covid 19, covid-19 india, extrapulmonary tuberculosis (eptb), genital tuberculosis

## Abstract

The COVID-19 pandemic has significantly impacted the global health system as well as the social and economic impact on tuberculosis (TB) treatment and diagnostic services. A high volume of patients diagnosed and treated for TB were impacted by the pandemic restrictions, particularly reduced access to TB services provided by the National Tuberculosis Elimination Programme in India; this in turn increased the number of deaths due to TB. The Indian healthcare system has been struggling with the eradication of TB, and this additional worldwide health crisis caused by SARS-CoV-2 has put the Indian healthcare system under severe stress. Both COVID-19 and TB are infectious diseases that primarily affect the lungs and have similar symptoms such as cough, fever, and difficulty breathing. The need of the hour is to take proper actions to mitigate and reverse these impacts urgently. The immediate priority is to aggressively step up the provision of essential TB services so that the levels of TB case detection and treatment return to at least pre-COVID-19 levels. The diagnosis of genital TB especially needs a high index of suspicion, as most of the cases are asymptomatic and diagnosed by chance in young women being evaluated for fertility. Here, we present a series of advanced genital TB cases that required intensive care and could have been detected and treated at an early stage.

## Introduction

Tuberculosis (TB) is a highly contagious infectious disease endemic in several developing regions of the world and is also the leading cause of mortality [1]. About 40% of the Indian population is infected with TB, with the vast majority having a latent TB infection. In 2020, the COVID-19 pandemic added to the stress of the already struggling Indian healthcare system. An extensive literature search shows enough evidence of worsening TB infections globally due to the COVID-19 pandemic. A COVID-19 infection results in immune dysregulation with clinical features ranging from asymptomatic to severe respiratory distress [2,3]. Similarly, immune system weakening may lead to an opportunistic infection or the reactivation of latent TB. Tuberculosis typically affects the lungs and multiple organs, and in a minority of patients, it may lead to respiratory failure and death [4].

The COVID-19 pandemic has undone years of progress in providing essential TB services in India. The reduced access to TB diagnosis and treatment during the pandemic has increased deaths. According to the WHO Global Tuberculosis Report 2022, about 1.6 million deaths occurred due to TB in 2021 (the estimate was up from 1.5 million in 2020 and 1.4 million in 2019) [5]. The declining rate of new TB infections achieved by our national TB program in previous years has come to a halt. There has been an increase in cases of advanced TB due to delays in treatment.

The need of the hour is to take proper actions to mitigate and reverse these impacts. The immediate priority is to aggressively step up the provision of essential TB services so that the levels of TB case detection and treatment revert to pre-COVID-19 levels. Many studies have found 20% to 53% of extra-pulmonary TB among all the cases of TB, and genital TB is seen in 9% of extra-pulmonary TB cases [6]. The latter is mostly asymptomatic and is diagnosed as a chance finding in young women being evaluated for fertility. In this case series, we report unusual presentations of advanced genital TB in the post-COVID-19 era.

## Case presentation

Case report 1

A 47-year-old para two, living two (P2L2) thinly-built woman with both normal deliveries presented to the casualty with complaints of severe lower abdominal pain. Her BP was 80/60 mmHg, and her pulse rate (PR) was 110/min. Her pain was sudden in onset and progressive in nature. There was a history of abdominal TB in her husband 15 years ago, which was fully treated. The patient's abdomen was soft and non-tender, and no abdominopelvic mass was felt. On speculum examination, the cervix and vagina were healthy, and no discharge was seen. Per the vaginal examination, the uterus was of normal size, anteverted, and there was fullness felt in the pouch of Douglas.

**Figure 1:**
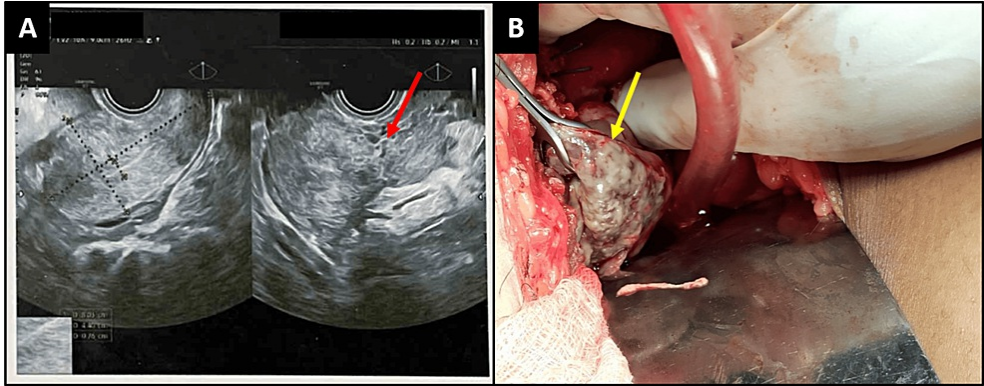
Ultrasound of the left adnexa shows the presence of heteroechoic mass lesion measuring 61x40x70 mm with internal necrosis (A). The intraoperative image reveals the left ruptured ovarian abscess measuring 6 cm x 7 cm (B).

The patient's hemoglobin was 12.4 g/dl, platelet count was 90,000/microliter, and WBC count was 12200/microliter. Ultrasonography revealed the uterus to be anteverted, normal in size, and with an endometrial thickness of 7 mm. The left adnexa showed the presence of a heteroechoic mass lesion measuring 61x40x70 mm, with internal necrosis and no definite sac-like structure or ring of vascularity on Doppler, but the presence of significant probe tenderness (Figure 1). The right ovary was normal. Other organs were within normal limits. Her culdocentesis yielded frank pus.

The patient was taken up for an emergency laparotomy because of deteriorating vitals. Around 800 ml of pus was present in the peritoneal cavity. The right-sided fallopian tube was inflamed, multiple tubercles were present on the surface, and the right ovary was normal. On the left side, adhesions were present between the uterus, tube, ovary, and bowel; the left tube was inflamed; tubercles were present over the tube; and an ovarian abscess (ruptured) of 6 cm x 7 cm was present on the left side (Figure 1). A left-sided salpingo-oophorectomy was done after adhesiolysis. The ovary had caseous necrosis on its surface. A right-sided salpingectomy was performed given the inflamed tube. Hemostasis was achieved, an abdominal drain was inserted, and the abdomen was closed in layers. There was an estimated blood loss of 400 mL. The report of pus as adenosine deaminase (ADA) was 142 U/L (normal range: 0-40 IU/L), and Ziehl-Neelsen (Z-N) staining for acid-fast bacilli (AFB) was negative. The Pus culture revealed no growth.

The patient was started on anti-tuberculosis treatment (ATT) on day 3 after the exploratory laparotomy on suspicion of abdominal TB. She was discharged from the hospital on day 10 and followed up in OPD for six months. She responded well to treatment. The histopathology report showed caseous necrosis and epitheliod cell granulomas in the left fallopian tube.

Case report 2

A 32-year-old gravida 2, para 1, living 1 (G2P1L1) thinly-built woman with previous normal vaginal delivery presented to our hospital with three months of amenorrhea and complaints of lower abdominal pain and bleeding per vaginum for five days. Her urine pregnancy test (UPT) was positive. On examination, her abdomen was soft and non-tender, with mild abdominal distension. The cervix and vagina looked healthy on a speculum examination. The vaginal examination revealed the uterus to be about eight to 10 weeks with fullness in the right fornix, and the uterus deviated to the left side. On culdocentesis, frank pus was aspirated.

**Figure 2:**
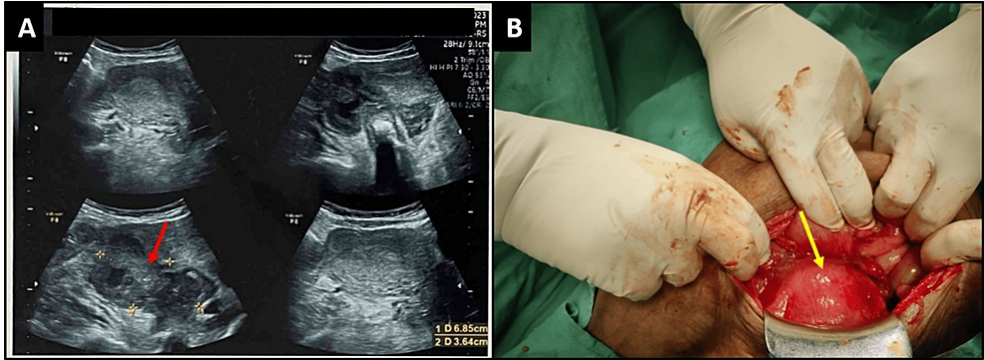
Ultrasound of the right adnexa shows a heteroechoic lesion of size 68 mm x 36 mm that was not separately seen from the right ovary (A). The intraoperative image reveals a bulky uterus with an edematous fallopian tube (B).

Her hemoglobin was 9.6 g/dl, her WBC count was 21900/microliter, and her erythrocyte sedimentation rate (ESR) was 62 mm in the first hour. Her platelet count was 529000/microliter. Her lactate dehydrogenase (LDH) was 696 U. On ultrasound, no gestational sac or products of conception were found. Endometrial thickness was 6.3mm. The right adnexa showed a heteroechoic lesion of size 68 mm x 36 mm, not separately seen from the right ovary (Figure 2). The left ovary was normal. Mild, free fluid was seen in the pouch of Douglas and the peritoneal cavity. 

The patient was taken for exploratory laparotomy as the patient had falling blood pressure. The uterus was enlarged to the eight-to-10-week size with the bowel adherent to the posterior wall of the uterus. Bilateral tubes were inflamed and adherent to the bowel and posterior wall of the uterus, making a localized abscess (Figure 2). While separating the bowel from the uterus, localized pus collection was seen, and a pus sample was sent for culture and sensitivity, Z-N staining, and ADA levels. Pus drainage and peritoneal washing were done. Left-sided hydrosalpinx was seen, and the ovary was unremarkable. The right-sided tube was beaded and had an edematous appearance. The fimbrial end of the fallopian tube and ovary could not be seen due to adhesions, and separation could not be done. The intra-abdominal drain was placed. As UPT was positive and tubal ectopic was ruled out, an incomplete abortion was suspected, and dilatation and curettage (D&C) were done. Only clots were removed, and no products of conception were present. The patient was shifted to the ICU due to persistent hypotension and hypoxia. She was on noradrenaline at 1 mL/hour. She was moved to the ward on day 7 and discharged on ATT on day 10. The pus culture sensitivity report showed mixed growth, and Z-N staining was negative. Pus ADA levels were 252 IU/L. The diagnosis of genital TB was made based on clinical and operative findings and the raised ADA levels of pus. The patient was followed up in the OPD for six months and responded well to treatment.

Case report 3

A 45-year-old para 4, live 4 (P4L4) well-built woman with previous normal deliveries presented to the casualty with complaints of acute pain in the abdomen and severe dysuria. The patient had a faintly positive UPT at home. Her BP was 120/78 mmHg, and her PR was 98/minute. There was no significant medical or surgical history. The general examination did not reveal any abnormalities. Her BMI was 23. On examination, her abdomen was soft and non-tender, with no pelvic-abdominal mass felt and no evidence of ascites. The cervix and vagina looked healthy on the speculum examination. On vaginal examination, the uterus was of normal size, with an adnexal mass of around 5 cm to 6 cm felt on the left side. The repeat UPT was negative.

The patient's hemoglobin was 11.0 g/dl, and her platelet count was 1.2 lakh/microliter. Her WBC count was 13,356/microliter. Her liver function test (LFT) and kidney function test (KFT) were within normal limits. Ultrasonography revealed an anteverted, normal-sized uterus. Endometrial thickness was 6 mm. The left adnexa showed the presence of a heteroechoic mass lesion measuring 56x30x60 mm. No free fluid was present. Her contrast-enhanced (CE) CT abdomen showed a left adnexal cystic lesion (63x28x65 mm, volume 160 cc) with multiple septations and without a solid component. Multiple subcentrimetric mesenteric lymph node enlargements were noted. A CT chest revealed a right lung middle lobe collapse. The kidney, liver, gall bladder, spleen, urinary bladder, ureters, and gastrointestinal tract (GIT) were unremarkable. The diagnosis of genital TB was made based on clinical and radiological findings. The patient was discharged from the ward on ATT medications. On follow-up visits at the OPD for six months, it was noted that the patient responded well to the treatment.

Case report 4

A 48-year-old P2L2 woman of average build who had normal vaginal deliveries presented to our hospital with complaints of severe lower abdominal pain with a BP of 90/60 mmHg and a PR of 118/minute. She did not have any significant medical or surgical history. There were no significant findings on the general physical examination. The abdominal examination revealed it to be soft, with no tenderness present and no pelvic or abdominal mass felt. On speculum examination, the cervix and vagina looked healthy. On the vaginal examination, the uterus felt bulky, about six to eight weeks in size, anteverted, bilateral fornices were free, and fullness was felt in the pouch of Douglas.

The patient's culdocentesis was positive, and frank pus was aspirated. Her lab profile values showed hemoglobin of 9.8 g/dl, WBC of 18000/microliter, platelets of 99000/microliter, and ESR of 68 mm in the first hour. Her LFT and KFT were within normal limits. Ultrasonography showed an anteverted uterus of normal size; the endometrial thickness was 8 mm. There was a pelvic mass with internal fluid echoes suggestive of a pelvic abscess.

The patient was taken up for exploratory laparotomy, which revealed 600 ml of pus present in the peritoneal cavity; this was drained and sent for investigations. The right-sided adnexa was adherent to bowel loops. The left-sided adnexa was inflamed. The bowel loops were adherent to the adnexa on the right side, adhesiolysis was performed, the right tube was inflamed, and tubercles were present over the tube. Pus ADA was 112 IU/L. The pus culture and sensitivity showed no growth, and Z-N staining for AFB was negative. The X-ray chest was within normal limits. The patient was started on ATT medications on postoperative day 3, discharged on postoperative day 5, and followed up for six months in OPD. She showed good signs of recovery as she responded well to treatment.

Case report 5

**Figure 3:**
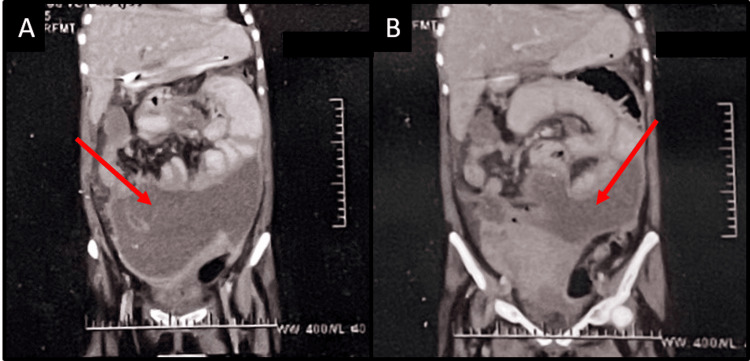
The CT of the abdomen (A and B) shows a large, peripherally enhanced collection intraabdominally anterior to the uterus and peritoneal thickening suggestive of peritonitis.

A 20-year-old para 1, live 1 (P1L1) patient with postnatal day 17 of normal vaginal delivery at a private hospital presented to the casualty of our hospital with the complaint of fever for 10 days, vomiting, and pain in the abdomen for five days. Her fever was insidious in onset, progressive in nature, more frequent during the evening, associated with shivering and relieved after taking medications. She also complained of pain in the abdomen for five days, which was sudden in onset, progressive in nature, aggravating with movements, and there were no relieving factors. Her vitals were as follows: BP 90/60 mmHg, PR 110/minute. She had an Hb of 8.3 g/dl, a platelet count of 96400/microliter, a WBC count of 25700/microliter, an AST of 62.9, an ALT of -68.5, and an ESR of 64 mm in the first hour. The chest X-ray was within normal limits. Ultrasonography revealed the liver enlarged by 16.2 cm, altered echotexture, rest within normal limits, and a moderate amount of fluid collection in the peritoneal cavity. Her CT abdomen showed a large, peripherally enhanced collection of 17x18x10 cm anterior to the uterus and extending intraabdominally. Peritoneal thickening was seen in the right paracolic gutter, suggesting peritonitis with peritoneal collection formation and paralytic ileus (Figure 3).

Pigtail catheterization was done, and 1400 ml of pus was drained. However, the patient subsequently had features suggestive of intestinal obstruction, and an exploratory laparotomy was done. Her intraoperative findings included 50 cc of necrotic collection that was aspirated, bowel adherent to the omentum, and bilateral adnexa that could not be visualized. Subhepatic and pelvic drains were inserted. The patient then shifted to the ICU. Her postoperative period was uneventful. The pus ADA was 178 IU/L, Z-N staining for AFB was negative, and the pus culture did not show any growth. The patient was discharged from the ward and started on ATT medication. On follow-up for six months in the OPD, the patient showed a good therapeutic response.

Table 1 summarizes all the patients mentioned in this case series.

**Table 1:** Summary of patients featured in the five case reports AFB: Acid-fast bacilli; Z-N: Ziehl-Neelsen; HPE: Histopathological examination; ADA: Adenosine deaminase; SpO2: Oxygen saturation; TB: Tuberculosis; P2L2: Para 2, live 2; G2L1: Gravida 2, para 1, live 1; P4L4: Para 4, live4; P1L1: Para 1, live 1

Characteristics	Case 1	Case 2	Case 3	Case 4	Case 5
Age (in years)/ gender	47/F	32/F	45/F	48/F	20/F
Parity	P2L2	G2P1L1	P4L4	P2L2	P1L1
History of TB	Contact with TB, 15 years back	No	No	No	No
History of COVID-19	Yes, 8 months prior to presentation	No	Yes, 3 months prior to presentation	Yes, 4 months prior to presentation	No
COVID-19 immunization	One dose	No	No	No	No
Pus aspirate ADA	142 IU/L	252 IU/L	NA	112 IU/L	178 IU/L
AFB seen Z-N staining	No	No	No	No	No
Procedure performed	Exploratory laparotomy	Exploratory laparotomy	No	Exploratory laparotomy	Exploratory laparotomy
ICU stay	Yes	Yes	No	No	Yes
Inotropes requirement	Yes	Yes	No	No	Yes
Oxygen required	Yes	Yes, SpO2 93%	No	No	Yes
CT abdomen findings	NA	NA	Left adnexal cystic lesion with multiple septations without solid component	NA	Large peripherally enhanced collection anterior to the uterus, peritoneal thickening seen in the paracolic gutter
HPE report	Caseous necrosis with the presence of epithelioid cell granulomas	No	No	No	No

## Discussion

The SARS-CoV-2 outbreak has jeopardized health systems and greatly affected socioeconomic parameters. With the global focus on fighting this unpredictable fight with this new virus, the biggest chronic infectious killer, Mycobacterium tuberculosis, was hugely affected by this shift in attention [7]. The largest number of TB cases are found in India and China. India accounts for a quarter of all drug-sensitive and multidrug-resistant TB (MDR-TB) cases. The WHO had aimed to eliminate TB by 2035, whereas India aimed to eliminate it by 2025. Strong measures were initiated, including strict notification, active case finding, and the 99 directly observed therapy, short (DOTS) course to ensure compliance with treatment. However,all these efforts to control TB were severely impacted by the COVID-19 pandemic. The Government of India imposed nationwide lockdowns multiple times during the sequential COVID-19 waves. It was a big challenge to ensure the smooth functioning of program services, leading to disruptions in routine TB care. The focus on managing the COVID-19 pandemic has strained health care systems, leading to disruptions in TB diagnosis and treatment services. 

An estimated 10.6 million people fell ill with TB worldwide in 2021, an increase of 4.5% from 10.1 million in 2020, reversing many years of slow decline. Similarly, the TB incidence rate is estimated to have increased by 3.6% between 2020 and 2021, following declines of about 2% per year for most of the past two decades. Globally, the annual number of deaths from TB fell between 2005 and 2019, but this trend was reversed in 2020 and 2021. In 2021, there were an estimated 1.4 million deaths among HIV-negative people and 187,000 among HIV-positive people, for a combined total of 1.6 million. This was up from the best estimates of 1.5 million in 2020 and 1.4 million in 2019, and reverted to the level of 2017 [6]. An increase in the number of people with TB who are not detected by health services results in an increase in the number of people with undiagnosed and untreated TB in the community. This may lead to an increase in the number of cases of advanced TB. In a study conducted in Spain by Ruiz-Bastián et al., it was observed that there was a 67.7% reduction in pulmonary TB diagnoses during the COVID-19 pandemic. However, at the same time, extra-pulmonary TB increased by 33.3% [8].

Globally, there were an estimated 450,000 incident cases of multidrug-resistant TB (MDR-TB)/rifampicin-resistant TB (RR-TB) in 2021, up 3.1% from 437,000 in 2020. There was an overall increase in TB incidence between 2020 and 2021, estimated to have occurred due to the impact of the COVID-19 pandemic on TB detection. An estimated 191,000 deaths occurred due to MDR/RR-TB in 2021 [5]. Tuberculosis after recovering from COVID-19 is becoming more common, potentially leading to a TB outbreak in the post-COVID-19 era [9]. The immunosuppressive nature of the disease and its treatment modalities may contribute to post-COVID-19 TB. There are case reports supporting the association between COVID-19-related immune suppression and the reactivation of TB, suggesting that COVID-19 might accelerate the progression of latent TB infection (LTBI) to a severe form of TB, even in mildly infected patients [10]. 

In our clinical practice in a tertiary-level center in Delhi, we witnessed a series of cases with upstaging of genital TB. The usual presentation of genital TB in our center is by chance detection during the evaluation of fertility or as an adnexal mass (hydrosalpinx). Sharma et al. reported 90% involvement of fallopian tubes in cases of genital TB [6]. Grace et al. also reported that most of the patients are asymptomatic and are usually diagnosed during evaluation for fertility [11]. However, all patients in the present series presented in critical condition with deteriorating vitals from either acute abdomen or abdominal collection, which is unusual.

During the year immediately after COVID-19 was declared to have ended, we witnessed a series of cases presenting as acute abdomen with the presence of pyoperitoneum, with findings consistent with advanced genital TB. Three cases were in the perimenopausal age group, and two were in the reproductive age group. Studies report that most cases of genital TB occur in fertile individuals between the ages of 20 and 45 [12]. Genital TB is known to cause infertility, but this was not corroborated in our case series. All the women in our case series of advanced genital TB had live children. So, it is likely that genital TB was a recent development in these patients. A recent history of COVID-19 infection was present in cases one, three, and four. Studies have shown that there is an increase in vulnerability to TB infection due to COVID-19. The COVID-19 infection has been shown to affect the innate immune system and T cells, which are essential to prevent LTBI reactivation. Also, exposure to COVID-19 can lead to T cell dysfunction, cytokine storms, and a reduction in lymphocytes, especially CD8+ and CD4+ T cells [13]. Four out of five patients required exploratory laparotomies and were admitted to the intensive care unit. Three of the patients required inotropic and ventilatory support. We observed that post-COVID genital TB had an atypical presentation and was severe. A history of COVID-19 infection combined with frequent lockdowns and movement restrictions and a strained healthcare system during the pandemic led to the worsening of TB in the patients featured in this case series. Healthcare resources were diverted away from other conditions, potentially leading to delays in the diagnosis and treatment of genital TB and advanced diseases, including pyoperitoneum in these patients.

Individuals who already suffer from HIV infection or any other form of acquired or innate immunocompromised state are more at risk of TB. Malnutrition, poor access to health care, and poverty are additional risk factors [12]. The presence of these immunosuppressive conditions has been seen to worsen TB. Critchley et al. reported that diabetes increases the risk of TB and poor TB treatment outcomes [14]. There were no comorbid conditions such as diabetes and HIV in our case series.

## Conclusions

In our tertiary care institute, we came across a series of advanced genital TB cases that were Z-N stain-negative but responded well to ATT medications. This empirical treatment is mainly based on clinical, laboratory, and intraoperative findings. Most of the patients required surgical intervention and intensive care because of deteriorating vitals. Their conditions could have been detected and treated at an early stage if there were no COVID-19 pandemic restrictions.
